# Clinical Factors Associated with Violence Behavior in Persons with Schizophrenia Spectrum Disorders: A Case Control Study

**DOI:** 10.1192/j.eurpsy.2022.882

**Published:** 2022-09-01

**Authors:** N. Zavradashvili, O. Toidze

**Affiliations:** 1 The University of Georgia, School Of Health Sciences, Tbilisi, Georgia; 2 East European University, Faculty Of Healthcare Sciences, TBilisi, Georgia

**Keywords:** schizophrénia, violence behavior

## Abstract

**Introduction:**

Study of clinical risk factors of violent behavior in persons with schizophrenia still remains actual in research literature. This is especially important for Georgia as ongoing mental health reform aims to shift from institutional care to community based services, which in turn requires better understanmding and management of risk of aggressive behavior in the community.

**Objectives:**

To study clinical risk factors for violence in persons with schizophrenia spectrum disorders (SSD) who have committed a violent act in the past and in persons with SSD who have not committed a violent act.

**Methods:**

The survey design was retrospective case-control study. Case-control groups were defined according to the outcome (violent act in the past). We studied the impact of clinical symptoms on each person using standardized scales for assessment of the positive and negative symptoms and global level of functioning. Data were collected through patient interviews and medical records.

**Results:**

Study results showed that diagnosis of delusional disorder and ideas of persecution were associated with increased risk of violence (28.7% cases versus 7.5% controls); Hallucinations if presented were less severe compared with controls (2.1% vs. 7.5%). Negative symptoms were marked in cases but more severe in controls. Of cases 43,6% showed serious impairment of global functioning (vs 25,5% controls).

**Conclusions:**

Study findings confirmed that a focus on improving controllable clinical factors, including global level of functioning, might help to prevent aggressive behavior. It is discussed that developments and implementation needs-specific services to reduce risk of violence behavior should be prioritized by mental health national strategy plan.

**Disclosure:**

No significant relationships.

